# Rejuvenating somatotropic signaling: a therapeutical opportunity for premature aging?

**DOI:** 10.18632/aging.100262

**Published:** 2010-12-26

**Authors:** Alejandro P. Ugalde, Guillermo Mariño, Carlos López-Otín

**Affiliations:** Departamento de Bioquímica y Biología Molecular, Facultad de Medicina, Instituto Universitario de Oncología, Universidad de Oviedo, 33006-Oviedo, Spain

**Keywords:** progeria, cancer, growth hormone, insulin-like growth factor, longevity

## Abstract

We have recently reported that progeroid Zmpste24^−/−^ mice, which exhibit multiple defects that phenocopy Hutchinson-Gilford progeria syndrome, show a profound dysregulation of somatotropic axis, mainly characterized by the occurrence of very high circulating levels of growth hormone (GH) and a drastic reduction in insulin-like growth factor-1 (IGF-1). We have also shown that restoration of the proper GH/IGF-1 balance in Zmpste24^−/−^ mice by treatment with recombinant IGF-1 delays the onset of many progeroid features in these animals and significantly extends their lifespan. Here, we summarize these observations and discuss the importance of GH/IGF-1 balance in longevity as well as its modulation as a putative therapeutic strategy for the treatment of human progeroid syndromes.

## Somatotropic regulation of aging

Although the maximal lifespan of an organism is probably genetically determined, the combined accumulative action of different types of stressors, such as oxidative damage, telomere attrition and the decline of DNA repair and protein turnover systems determines the real lifespan and contributes to the so-called aging process [1,2]. Currently, the dynamics of aging is far from being completely understood, but our knowledge of the molecular basis of this complex process has considerably increased in recent years. Among the different processes and pathways involved in lifespan regulation, GH/IGF somatotropic signaling has gained relevance as one of the major determinants of longevity in different and evolutionary distant species, including mammals [3,4]. The pivotal role of somatotropic axis in the regulation of lifespan was first shown by Kenyon and colleagues, who observed that mutation of specific genes essential for IGF-related signaling was able to considerably extend longevity in nematodes [5]. Currently, it is widely accepted that the down-regulation of IGF-related signaling extends lifespan in a wide variety of model organisms and it is also a common feature of long-lived individuals [6,7].

Almost two decades after the first evidence on the somatotropic axis role in lifespan regulation, many other signaling pathways and metabolic routes have been shown to participate in the aging process [8-14]. Remarkably, some of these advances in the understanding of aging have been boosted by the study of both accelerated aging syndromes and progeroid animal models [15-17]. Progeroid syndromes are dramatic diseases in which certain features of human aging are prematurely developed, leading to a considerable reduction in the lifespan of patients, although the specific features and impact on longevity are highly dependent on the molecular nature of the disease. Many progeroid syndromes are caused by defects in DNA repair mechanisms but others derive from defects in nuclear envelope maintenance [18-21]. In fact, mutations in the *LMNA* gene encoding lamin A -a fundamental constituent of the nuclear envelope- or in that encoding the metalloproteinase ZMPSTE24 involved in the proteolytic processing of lamin A- cause dramatic human progeroid syndromes, such as Hutchinson-Gilford progeria syndrome (HGPS), atypical Werner syndrome, mandibuloacral dysplasia and restrictive dermopathy [20,22,23]. The clarification of the molecular mechanisms underlying these devastating diseases has been impulsed by studies of *Lmna*- and *Zmpste24*-deficient mice, which show nuclear architecture aberrations and a number of histopathological alterations that phenocopy human syndromes of accelerated aging [24-26].

**Figure 1.**
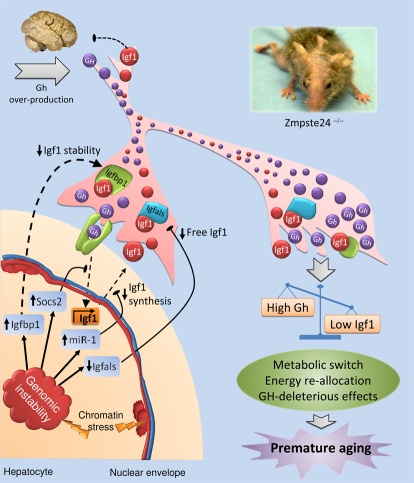
Proposed model for the somatotroph axis alterations of *Zmpste24^−/−^* progeroid mice. Nuclear envelope abnormalities present in *Zmpste24*-deficient cells cause chromatin detachment from the nuclear envelope and a profound structural disorganization. This genomic instability activates a chronic DNA damage response, which in turn triggers an adaptive response aimed at re-allocating resources from growth to somatic preservation. Hepatocytes are involved in this adaptive response through its ability to modulate the somatotropic axis and respond to the chronic DNA damage altering the expression of some key genes that affect Igf1-signaling at several levels. First, the reduced growth-hormone receptor (Ghr) transcription and the up-regulation of suppressor of cytokine signaling-2 (Socs2), diminishes the Gh-mediated Igf1 transcription. In addition, miR-1 over-activation represses Igf1 synthesis, reinforcing the already reduced Igf1 transcription and compromising the circulating levels of Igf1. The availability and stability of free Igf1 is reduced through up-regulation of insulin-like growth factor binding protein-1 (Igfbp1) and down-regulation of Igfbp-acid labile subunit (Igfals), respectively. Consequently, Igf1-signaling is diminished in the whole organism, which in turn leads to a metabolic switch towards somatic maintenance at the expense of somatic growth. In parallel, the low levels of Igf1 fail to inhibit Gh secretion at the pituitary gland, generating the high levels of circulating Gh present in these progeroid mice, which could aggravate the situation through its deleterious effects. Dotted lines represent disrupted pathways and blunt arrows indicate inhibitory actions.

## Somatotropic signaling in progeria

Given the prominent and evolutionarily conserved role of IGF-1-related signaling in lifespan regulation, we have recently examined the somatotropic axis in mice deficient in the Zmpste24 metalloproteinase, which present a strong progeroid phenotype and exhibit many features observed in HGPS patients [25]. We have found that *Zmpste24^−/−^* mice show a profound dysregulation of GH/IGF-1 balance, mainly characterized by the progressive reduction of blood IGF-1 levels, the progressive increase in circulating GH levels and the generation of marked transcriptional alterations in key genes for somatotropic signaling [27]. In fact, genes coding for proteins involved in IGF-1 stability or in IGF-1 signaling - such as *Igfbals, Ghr, and Igf1*itself- are transcriptionally down-regulated in livers from *Zmpste24*-deficient mice. Conversely, genes encoding proteins involved in IGF-1 signaling suppression such as *Igfbp1*,*Socs2* and *Socs3* are significantly upregulated in progeroid mice, especially in the case of *Igfbp1*, whose mRNA levels are more than 50-fold higher as compared with wild-type mice values. The finding of these marked alterations of somatotropic axis components in *Zmpste24^−/−^*mice, and the observation of reduced *Igf1* mRNA and protein levels even in the presence of high circulating GH levels, was indicative of a GH resistance process similar to that described in some HGPS patients[28]. However, after analyzing the status of the main transduction pathways activated by GH in response to intravenously injected recombinant GH, we excluded the possibility that the transcriptional down-regulation of *IgfI* observed in *Zmpste24^−/−^*mice is caused by a liver-specific GH-resistance derived from signal transduction abnormalities in archetypal pathways [27].

Based on these findings, we next explored different possibilities which could explain the observed decrease of *Igf1*mRNA levels in progeroid mice, assuming that this should be the initial and major event accounting for the dysregulation of somatotropic axis found in *Zmpste24^−/−^* progeroid mice [27]. After an exhaustive search for alterations that could be responsible for the observed reduction in *IgfI* mRNA, we found that miR-1 -a microRNA predicted to target *IgfI*- was significantly upregulated in a variety of tissues from *Zmpste24^−/−^*mice. Interestingly, we also observed that this micro-RNA was significantly upregulated in fibroblasts from HGPS patients, which also show profound nuclear envelope abnormalities. Since these experimental findings suggested a causal relationship between nuclear envelope defects and miR-1 upregulation, we decided to characterize in more detail the putative relevance of miR-1 as a specific microRNA involved in *Igf1* control. After performing a series of specific luciferase assays, usually employed for microRNAs target validation, we could confirm that the 3'-UTR of *Igf1* contains a functional binding site for miR-1, thereby demonstrating that miR-1 is a *bona fide* microRNA targeting *Igf1* regulator [27,29]. Accordingly, it is tempting to speculate that the characteristic nuclear lamina abnormalities present in both HGPS and *Zmpste24^−/−^* cells induce -by a yet unknown mechanism- an upregulation of miR-1, which in turn leads to a reduction in *Igf1* mRNA cellular content and finally, to a decrease in circulating IGF-1 (Figure 1). As a consequence of this decrease, blood GH levels start to rise in an attempt to increase IGF-1 synthesis. However, and although GH is able to stimulate the *IgfI* mRNA transcription in progeroid cells, the increased levels of miR-1 may contribute to block IGF-1 protein synthesis. Taken to-gether, all these events may initially lead to the observed dysregulation in the essential GH/IGF-1 balance, which in turn probably accounts for all the other alterations in somatotropic axis components found in *Zmpste24^−/−^* progeroid mice (Figure 1).

To test this hypothesis and to evaluate whether a restoration of circulating IGF-1 levels in *Zmpste24^−/−^* mice could have a positive effect on the lifespan and progeroid features of these prematurely aged mice, we treated mutant mice with recombinant IGF-1 through the use of osmotic pumps which allowed a constant and stable delivery of the recombinant protein to bloodstream. Surprisingly, exogenous administration of IGF-1 not only restored the GH/IGF-1 balance, but also significantly extended mutant mice lifespan and ameliorated or delayed the onset of many of their progeroid features [27]. These findings strongly suggest that the dysregulation of somatotropic axis observed in *Zmpste24^−/−^* progeroid mice is a detrimental phenomenon, rather than a successful adaptive strategy.

## Concluding remarks and perspectives

Although most of the physiological and pathological features of progeroid individuals resemble those found in aged individuals, recent studies on prematurely-aged mice have facilitated the identification of surprising and paradoxical similarities between progeria patients and individuals with exceptional lifespan [30,31]. Among these similarities, the fact that circulating IGF-1 levels of progeroid mice are similar to those observed in long-lived or calorie-restricted individuals is remarkable, given the profound implications of IGF-1 signaling in lifespan regulation. Apparently, these paradoxical similarities between prematurely-aged and long-lived animals could derive from an adaptive effort to adjust the values of many biologic parameters to those which are compatible with a maximal lifespan, perhaps in an attempt to slow down the onset of premature aging. However, the precise physiological significance of these surprising alterations has not been studied in detail so far. It seems that this kind of metabolic and hormonal reprogramming is elicited when DNA damage or chromosomal instability signals reach a certain threshold. In fact, similar changes to those observed in prematurely-aged, calorie-restricted or long-lived individuals in terms of somatotropic axis dysregulation are seen in wild-type mice subjected to chronic oxidative or genotoxic stress [32-34]. It seems reasonable that in these conditions, which probably mimic those present in progeroid mice, a massive cellular induction of checkpoint and anti-proliferative responses would lead to a general endocrine and metabolic shift at the organismal level. This type of metabolic reprogramming would reallocate resources from growth to somatic preservation in an attempt to reduce replication defects, chromosomal instability, nuclear envelope abnormalities and finally, the risk of developing cancer [31]. Hence, in transient situations of excessive genotoxic damage, a systemic metabolic reprogramming towards preservation rather than proliferation would be of pivotal importance in order to survive until problems are solved. Similarly, during physiological aging, DNA damage accumulation would progressively induce a similar endocrine and metabolic shift -although in a much slower fashion- that is only observed in long-lived individuals who have not succumbed to cancer or to any other age-associated diseases.

A similar situation likely applies to individuals subjected to calorie restriction regimes, in which nutrients scarcity and the subsequent reduction of available ATP, would be the driving forces accounting for a general metabolic reprogramming which would contribute to extend longevity. However, if anti-proliferative signals are too strong and prolonged in time, as presumably occurs in premature aging syndromes, a chronic overactivation of this initially protective reprogramming could be detrimental for the organism, leading to a systemic senescent state and finally, to premature death. In this sense, the somatotropic axis alterations found in *Zmpste24^−/−^* progeroid mice represent an example of over-suppression of a growth-promoting pathway, as they show multiple and probably redundant alterations in regulators of GH-mediated signaling. In addition, the observed upregulation of miR-1 likely contributes to the exacerbated suppression of liver IGF-1 synthesis, even in the presence of high circulating GH levels. In fact, it is possible that these elevated levels of circulating GH could directly elicit detrimental responses in peripheral tissues, which could contribute to the development of accelerated aging. Interestingly, conditional mutant mice with liver-specific *IgfI*-deficiency have reduced IGF-1 levels and increased GH circulating levels, as well as a series of concomitant metabolic defects that can be ameliorated by treatment with a GH antagonist [35]. Moreover, giant GH transgenic mice exhibit several characteristic features of premature aging [36]. Accordingly, it is reasonable to speculate that an initially protective response involving a down-regulation of IGF-1 signaling may lead to detrimental effects when it is chronically overactivated and causes a profound dysregulation of somatotropic axis.

Our finding that restoration of somatotropic balance by recombinant IGF-1 administration not only ameliorates several progeroid features of *Zmpste24^−/−^*mice, but also extends their longevity, strongly supports this hypothesis. Consequently, and although an uncontrolled IGF-1 treatment could lead to the massive blockade of the anti-proliferative responses characteristic of *Zmpste24^−/−^*mice and favor cancer development, a selective modification of some altered signaling pathways could be beneficial for extending longevity without increasing the risk of malignancies. In this regard, it is remarkable that a reduction in p53 signaling increases lifespan and delays the onset of progeroid features in *Zmpste24^−/−^*mice [26]. Thus, given that somatotropic axis status is altered in some types of progeria and that recombinant IGF-1 treatment is effective for treating GH-refractory somatotropic alterations without significant detrimental effects [37], it is reasonable to consider recombinant IGF-1 treatment, alone or in combination with other drugs [38], as an experimental therapeutic strategy for children with progeria. Further studies with improved murine models of human progeria [16] will be necessary before this possibility can be translated into a clinical reality.
